# Fingertip Fiber Optical Tactile Array with Two-Level Spring Structure

**DOI:** 10.3390/s17102337

**Published:** 2017-10-13

**Authors:** Jelizaveta Konstantinova, Agostino Stilli, Kaspar Althoefer

**Affiliations:** 1 Advanced Robotics @ Queen Mary (ARQ), Faculty of Science and Engineering, Queen Mary University of London, London E1 4NS, UK; k.althoefer@qmul.ac.uk; 2 Centre for Medical Imaging Computing, University College of London, Gower Street, London WC1E 6BT, UK; a.stilli@ucl.ac.uk

**Keywords:** tactile sensing, force sensing, grasping

## Abstract

Tactile perception is a feature benefiting reliable grasping and manipulation. This paper presents the design of an integrated fingertip force sensor employing an optical fiber based approach where applied forces modulate light intensity. The proposed sensor system is developed to support grasping of a broad range of objects, including those that are hard as well those that are soft. The sensor system is comprised of four sensing elements forming a tactile array integrated with the tip of a finger. We investigate the design configuration of a separate force sensing element with the aim to improve its measurement range. The force measurement of a single tactile element is based on a two-level displacement that is achieved thanks to a hybrid sensing structure made up of a stiff linear and flexible ortho-planar spring. An important outcome of this paper is a miniature tactile fingertip sensor that is capable of perceiving light contact, typically occurring during the initial stages of a grasp, as well as measuring higher forces, commonly present during tight grasps.

## 1. Introduction

The problem of tactile perception for grasping along with the development of robotic hands has been extensively studied in recent years. Finding solutions for tactile perception is critical for the development of multi-fingered robotic hands capable of performing human-like grasping. Sensory information from the fingers and fingertips for robotic hands can provide essential information to improve the quality of a grasp, especially in terms of stability, robustness and the relative configuration between fingers. Many current grasping algorithms use vision only to plan the grasping strategy; however, it has been shown that tactile perception can significantly improve the performance, especially in situations where the view from the external vision system is occluded [1].

The use of tactile technologies integrated into multi-fingered robotic hands has been implemented using various approaches. Sensing devices can be the integral part of the structure of the fingertip or can be added to the finger in the form of a thin, skin-like structure [2]. A thin sensing structure added on the fingertip, typically comprises a tactile array, such as in [3,4]. A sensing array allows perceiving the points of contact, as well as normal forces. However, capacitive or piezoresistive sensors that are typically used as distributed sensors suffer from temperature and electrical noise dependence, nonlinearity and hysteresis [5]. Integrating a tactile or force sensor with a fingertip provides the benefit of measuring multi-dimensional forces, force vector and moments at the point of interest [6,7,8]. Such integrated approaches usually also allow detection of the point of contact, as shown in [9].

Although the design of a tactile system can be very application-specific, it is possible to outline the general requirements for such an approach. This includes broad sensing range, high sensitivity parameters, miniature size, and robustness to electric noise. In addition, it is desirable to perceive tactile information at multiple points by using multi-element sensor arrays.

**Figure 1:**
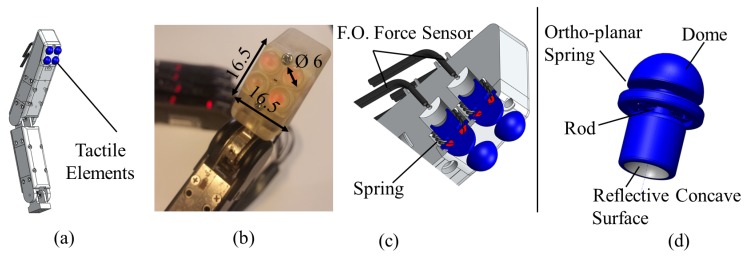
Fibre optical fingertip tactile sensor: (**a**) design of the fingertip fiber optical sensor; (**b**) photo of sensor integrated with prototype fingertip, dimensions are shown in mm; (**c**) section view of the fingertip sensor: fingertip tactile array; (**d**) hemispherical dome-shaped element.

The use of fiber optics in sensing is beneficial for a number of positive aspects, such as the capability to create miniature, lightweight and low-cost sensing structures with a large measurement bandwidth, immunity to electric noise and magnetic interference [10,11]. In this work, we present a tactile sensor system that can be integrated with the tips of robot fingers, as shown in Figure 1a,b. Exploiting the advantages of optical fibres for sensing (including the desired requirements of the sensor design for robotic hands), we focus on the use of fiber optical technologies for the estimation of applied forces commonly occurring during a grasp.

**Figure 2:**
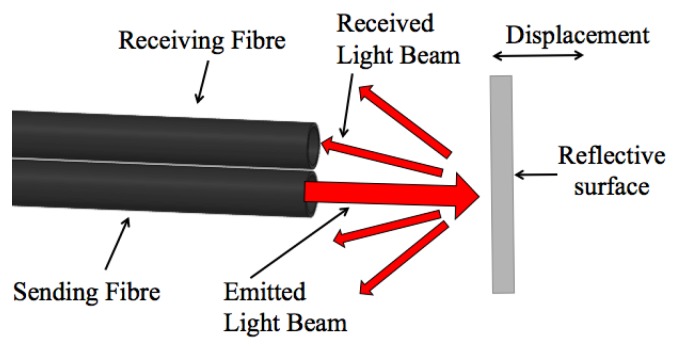
Measurement of the change of light intensity modulation using optical fiber.

The use of fiber optical technologies for force detection is becoming popular, and has shown its effectiveness for various applications [12]. One approach is to determine the force by measuring the change of light intensity varying as a function of the applied force. This can, for example, be achieved by deflecting a light-transmitting fiber and measuring the output light intensity [13,14]. Alternatively, it is possible to use a couple of fibers (that is, transmitting and receiving fibers) combined with a deformable mechanical structure with integrated reflective surface—then, the intensity of the reflected light is a function of the variable distance between the reflective surface and the receiving fiber, as shown schematically in Figure 2. For instance, sensors described in [15,16] are based on the displacements of a reflective surface. Another option is to use a bundle of optical fibers and to detect changes in reflected light using an external light source and a camera. Based on this force measurement principle, a tactile array sensor was developed in [17], as well as in [18].

The sensing concept described here is generic and is suitable for integration with the fingertips of various robotic hands. Our motivation is to develop an enhanced perceptual capabilities of a multi-fingered robotic hand mounted on a mobile manipulator for the very demanding, physical interaction with children in a kindergarten environment. The proposed sensing system was developed taking into account the advantages and shortcomings of earlier approaches [19], as well as taking into consideration the need to detect small forces, occurring during low-force interactions with objects of different stiffness, whilst at the same time being capable of measuring forces over a wide range. The implementation of a highly accurate capacitive sensor that detects low and high forces with high accuracy at a broad range was presented in [20]. However, this sensor requires complex manufacturing and calibration procedure. Based on our motivation, the proposed fingertip tactile sensor system has the following features:

- Tactile elements are to be organized in the form of an array on the area of the fingertip that will be in contact with the grasped object;
- Each single tactile element is sensitive enough to respond to low-force object interaction using the displacement of the ortho-planar spring (first level of displacement);
- Each single tactile element is also able to respond to higher forces that occur during a firm grasp using the displacement of the linear spring (second level of displacement).

In this paper, we explore the parameters and performance of linear and ortho-planar springs that are used to displace the reflective surface modulating that the light received by the receiving light in our tactile sensor elements. Further on, Section 2 describes the design and measurement principle of the sensing system. Section 3 describes finite element simulations. In Section 4, we present the performance of the tactile element using a linear spring and ortho-planar spring separately, and evaluate the combined response of a separate sensing element as well as the overall fingertip sensor performance when in contact with unknown objects. In Section 5, we draw our conclusions.

## 2. Measurement Principle and Design of the Fingertip Sensor

### 2.1. Description of the Design

The overall design of our array sensor integrated with a finger is shown in Figure 1. Four force sensing elements are composed of an array of tactile sensing elements. Fiber optic technology allows a good level of miniaturization, and, hence, a compact design. In the case of our prototype, the four sensing elements occupy an area less than 2.6 cm${}^{2}$ of the surface of the fingertip and a volume of 4 cm${}^{3}$.

**Figure 3:**
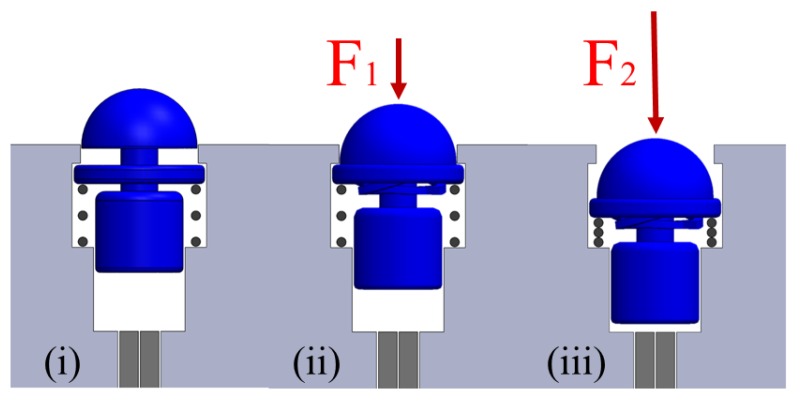
Compression of the tactile element: (**i**) no force is applied, the tactile element is not compressed; (**ii**) small force ($F_{1}$) is applied, ortho-planar spring only is compressed; (**iii**) large force ($F_{2}$) is applied, both ortho-planar and linear springs are compressed.

Each force sensing element of the tactile array consists of two fibers in a couple, as shown in Figure 3, to measure the light intensity modulation in response to applied forces and a hemispherical dome-shaped element, containing also the reflective surface. A linear spring is used to achieve translation of the dome-shaped element. The section view of the tactile array and a separate dome-shaped element is shown in Figure 1c,d. The dome-shaped element in the compressed state is shown in Figure 3. The rigid hemispherical element has been manufactured with a high resolution 3D printer (Project HD-3000 Plus, 3D Systems, USA). This technique is convenient and is suitable for the creation of miniature prototype structures. The characteristics of the plastic material used to fabricate the element are as follows: elastic modulus −1.283·10${}^{9}$ N/m${}^{2}$, Poisson’s ratio −0.94 N/A, shear modulus −3.189·10${}^{8}$ N/m${}^{2}$, mass density 1020 N/m${}^{2}$, and tensile strength is 4.24·10${}^{7}$ N/m${}^{2}$.

The hemispherical dome-shaped element (Figure 1d) is composed of four main sections: (1) a dome (6 mm in diameter), (2) an ortho-planar spring integrated in the tip of the dome, (3) a cylindrical rod and, and (4) a concave surface with reflective coating. The dome section is the part that interacts with the environment during grasping. Ortho-planar and linear springs are organized in series and act sequentially. The ortho-planar spring provides the first level of displacement of the rod. The second level of displacement is achieved using a linear spring. Both the ortho-planar and linear springs are used to provide the reaction force to return the sensing element to the initial position after the interaction with an object. The work in [21] has shown that the performance of this class of sensors can be enhanced using a concave reflective surface producing a reflected beam of higher light intensity at the point of the tip of the receiving fiber, when compared to the standard planar mirror. Hence, the inner surface of the sensing elements is shaped as a parabolic surface; a thin layer of silver reflective paint was applied on the 3D printed surface of the sensor structure to achieve the effect of reflection and beam forming. The reflective surface is not subjected to mechanical stress; hence, any mechanical deterioration of the applied paint layer, such as cracking or peeling off is avoided.

The pair of optical fibers is placed orthogonaly under the reflective surface, as shown in Figure 1c,d. As shown in the section view (a) of the same figure, the open terminals of the pair of optic fibers are placed orthogonaly under the reflective surface. When the sensor is not interacting with the environment, the distance between the fiber terminals and the reflective surface is 5 mm. A KEYENCE fiber optic transducer (FS-N10 series, Japan) is used to produce the light beam and to measure the light intensity of the reflected beam. This KEYENCE transducer converts the light intensity information into a voltage signal in the range of 0 to 4 V. The transducer allows for selecting power response mode that allows for detecting certain range certain range of light modulation intensity. Therefore, our system is setup only to detect larger light modulations that occur due to displacement of the reflective surface, and to filter smaller changes of light. This approach also eliminates the need for a reference fiber.

### 2.2. Design and Mathematical Description of the Ortho-Planar Spring

**Figure 4:**
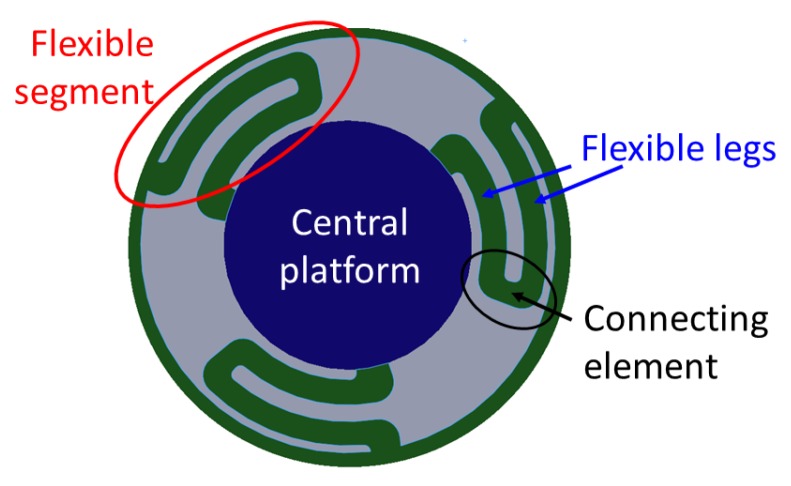
Ortho-planar spring: bottom view of the ortho-planar spring showing the structure of the flexible segments.

To ensure an additional level of displacement of the force sensing tactile element, an ortho-planar spring mechanism was used. The name of the spring comes from Greek “ortho”, which means straight. The benefit of such an approach is its compact flat design requiring little space; it is also easy to fabricate. In [22], the use of ortho-planar springs for force detection was successfully demonstrated. Ortho-planar springs can have different configurations according to the number of flexible segments (Figure 4). The minimum possible number of segments is two, in order to align the structure in parallel above the spring with the structure below the spring. The design of structure of the ortho-planar spring should be flexible enough to achieve displacement, and, at the same time, robust in order to avoid breaking. It is reported [23] that four segments do not provide better stability of the structure compared to three legs. Constructing an ortho-planar spring from a higher number of flexible segments is possible, but with a keen interest to achieve a miniaturized sensing structure, we chose a three-segmented approach.

We evaluate the parameters of the ortho-planar spring elements to show how they influence the spatial deflection of the overall structure. The deflection of a single flexible leg $\delta_{l}$ can be expressed again from the rectangular beam equation [24], considering small displacements, as:

(1) $$\delta_{l}=\frac{FL^{3}}{12EI},$$

where *F* is the applied force, *E* is the Young’s or elastic modulus, *I*-moment of inertia applied to each leg, and *L* is the length of the flexible leg. The elastic modulus of the material used for the fabrication of the spring is equal to 1.283·10${}^{9}$ N/m${}^{2}$, and the length of one flexible segment is 2.5 mm. The total deflection of the platform $\delta_{pl}$ is twice as big as the deflection of a single leg:

(2) $$\delta_{pl}=2\cdot \delta_{l}.$$

Hence, the deflection of the central platform and consequently of the reflective surface of the tactile element is:

(3) $$\delta_{pl}=\frac{FL^{3}}{6EI}.$$

In our design, each flexible spring element is made up of a rectangular beam with cross-sectional parameters—width *b* and height *h*. The moment of inertia for such a rectangular beam is:

(4) $$I=\frac{bh^{3}}{12}.$$

Combining Equations (3) and (4), it can be seen that the deflection of the platform is the function of the applied force and the following design parameters: Young’s modulus, *b*, *h* and *L*:

(5) $$\delta_{pl}=f\left\{F,L^{3},E,\frac{1}{b},\frac{1}{h^{3}}\right\}.$$

It can be seen that the platform’s small-scale deflection depends on the applied force, and on the material and geometric parameters of the ortho-planar spring. The Young’s modulus of the ortho-planar spring is fixed in our case, as it depends on the used 3D printing material. The length and width of the flexible legs are constrained by the design and overall dimensions of the spring. Therefore, the height *h* is chosen to be the variable parameter in our study, allowing us to adjust the performance of the ortho-planar spring, as shown in Section 4.2. In this work, we are interested to select the most appropriate configuration of ortho-planar spring, which can be used to detect light contact. The evaluation of spring parameters is shown below.

## 3. Finite Element Modeling of the Sensing Element Based on Ortho-Planar Spring

**Figure 5:**
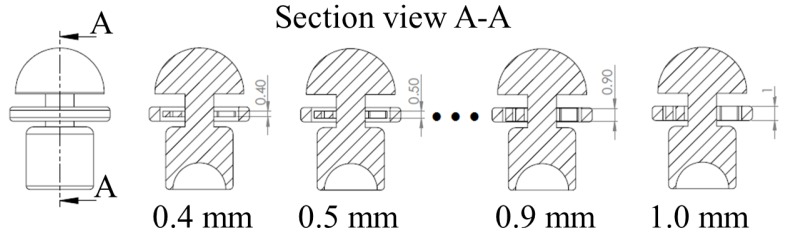
Design of the dome-shaped element exploring different spring leg thicknesses.

In order to select the most appropriate design parameters of the ortho-planar spring, finite element (FE) simulations have been performed. In addition, the information obtained in this section can be used as a reference for similar sensing systems that intend to employ the use of ortho-planar springs. As it is shown in the section above, the flexibility and stiffness of the ortho-planar spring depends on the thickness (height) *h* of the flexible leg of the spring, as shown in Figure 5. The size of the flexible leg was varied from 0.4 mm to 1 mm in order to determine the best configuration for the tactile element. Any dimension smaller than 0.4 mm results in a breakage of the spring, and dimensions above 1 mm are not suitable due to size limitation.

**Figure 6:**
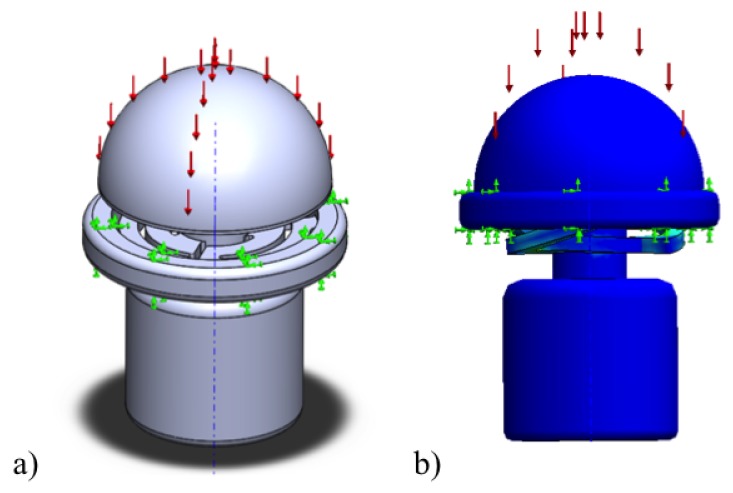
FE simulation of loading of ortho-planar spring: (**a**) Three-dimensional computer model with fixture points shown in orange; (**b**) simulation of the compressed spring.

The maximum displacement of the ortho-planar spring is 1 mm. A fully compacted spring under load is shown in Figure 6. During FE simulations, we have tested the force range that requires moving the spring to the maximum displacement. In the simulations, the tactile element was set fixed according to real conditions, as it is indicated in Figure 6. The FE simulations were run from zero force until the force that is needed to compact the spring, and the force step of 0.1 N was used. For the simulation purposes, it was assumed that the load is applied uniformly on the top of the tactile element. As a result of the simulations, normal displacement of the tactile element was recorded.

**Figure 7:**
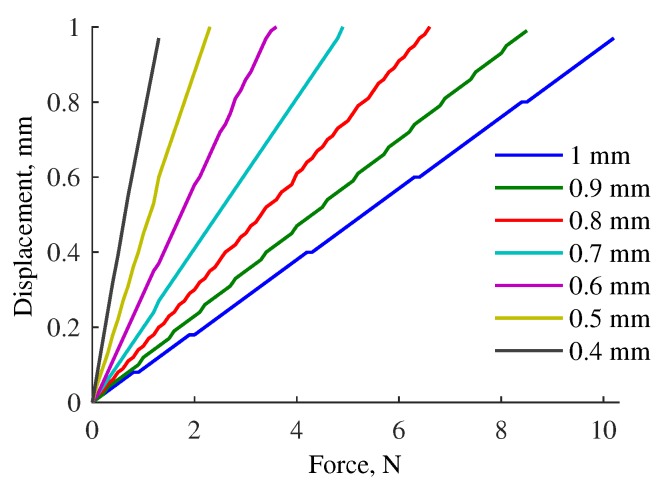
Force–displacement relationship obtained for different configurations of ortho-planar springs.

Figure 7 shows the relationship between the applied force and displacement of the ortho-planar spring. In our studies, we are particularly interested in measuring small forces that occur during light contact. However, in order to select the most suitable configuration of the ortho-planar spring, it is first required to analyze the force measurement performance of the linear spring separately. The ortho-planar spring with suitable parameters will be tested with loading and unloading mechanical tests.

## 4. Experimental Evaluation of the Performance of the Force Sensing Element

The next step of the evaluation of sensing element is to perform experimental analysis that include loading and unloading of the tactile elements. The experiments include the following stages:

1. As a first stage of experimental evaluation, the loading and unloading cycles are performed for the tactile element with the linear spring only. A tactile element without integrated ortho-planar spring was fabricated for these experiments.
2. As a second step of experimental validation, the calibration of the response of the ortho-planar with selected parameters based on the results of FE modeling is performed.
3. Finally, the combined performance of the tactile element incorporating linear and ortho-planar springs is assessed.

Sensor loading and unloading is used here to define the relationship between the physical measurement value (force) and the corresponding output voltage. The obtained relationship is represented using a mathematical equation describing a calibration curve. To estimate how accurately the chosen equation is representing the response of the sensor, the R-square value is evaluated between the calibration curve and the measured data. The calibration curve is chosen based on the best fit.

**Figure 8:**
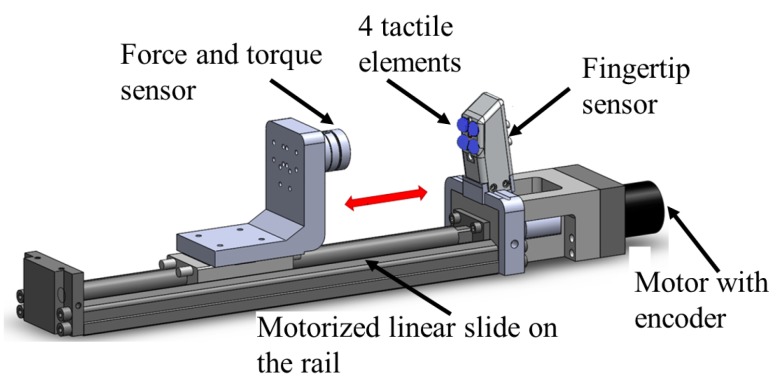
Calibration setup comprised from the linear motorized slide to implement repetitive movement, and a force and torque sensor to measure control forces.

In order to perform calibration of the force sensing element, loading and unloading cycles were performed. A motorized linear slide with defined motion was used for this purpose (Figure 8). The modulation of light intensity caused by the displacement of the reflective surface and the resulting voltage were recorded along with the force readings from a ground-truth force and torque sensor— in our experiments, we used the NANO17 from ATI technologies (Canada) to calibrate and benchmark our sensors. The ground-truth sensor was used to apply and measure the interaction forces from a single tactile element for the calibration and characterization of the linear and ortho-planar springs separately. The movement of the linear slide was performed at a constant speed (0.25 mm/s) to load and unload the force sensing element. Twenty loading and unloading cycles were performed for each set of parameters to test the characteristics the force response, as well as to outline the calibration curve. In order to avoid any effects from a previous cycle, the slide is paused for five seconds before the next cycle commences. Voltages from the KEYENCE converters were recorded at a frequency of 1 kHz using a National Instruments (TX, USA) data acquisition (DAQ) card.

### 4.1. Characteristics of the Force Sensing Element Based on Linear Spring Displacement

Firstly, it is required to evaluate the performance of the tactile elements using linear springs with different spring rates. Based on the performance of the previous prototype [19], where the parameters of the spring were assigned based on empirical evaluation, in this study, we tested springs with ratios 0.05, 0.1 and 0.2 N/mm. The spring rate is the parameter describing the amount of force that is required to compress a spring to a unit distance measure. The performance of the sensor can be described by sensing range, sensitivity and associated resolution. The maximum force was calculated using the reading of the ground-truth sensor, which corresponds to the maximum reading before saturation of the tactile element. Sensitivity was estimated as a median ratio between the output voltage of the tactile element until saturation (measured by KEYENCE) and force values detected by the ground-truth force sensor. The resolution of the proposed tactile element can be calculated dividing the resolution of KEYENCE converter (0.01 V) by the sensitivity of the tactile element.

Characteristics obtained for tactile elements with different spring constants are shown in Table 1. The tactile element with a maximum spring rate of 0.2 N/mm has a lower sensitivity, but has a wider force sensing range.

**Figure 9:**
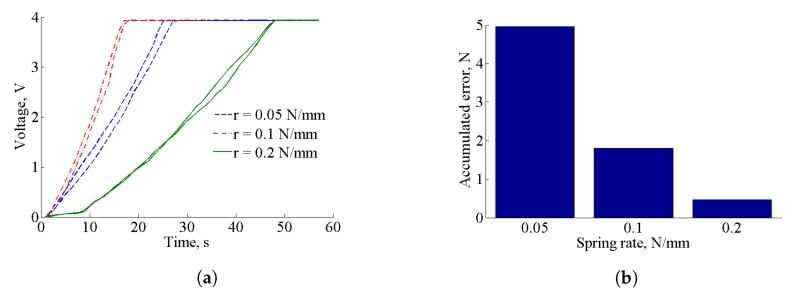
Performance of the hysteresis for liner springs. (**a**) Hysteresis curves for force elements with different spring rates; (**b**) Accumulated errors for hysteresis loops for force elements with different spring rates.

The additional parameter that describes the performance of the force sensor is the performance of it during loading and unloading cycles, i.e., hysteresis, as shown in Figure 9a. The minimal accumulated error due to hysteresis appears for the tactile element with a spring ratio of 0.2 N/mm (Figure 9b). It is calculated as a difference between loading and unloading cycles for tactile elements with corresponding springs, and normalized by the maximum detectable force for each spring.

**Figure 10:**
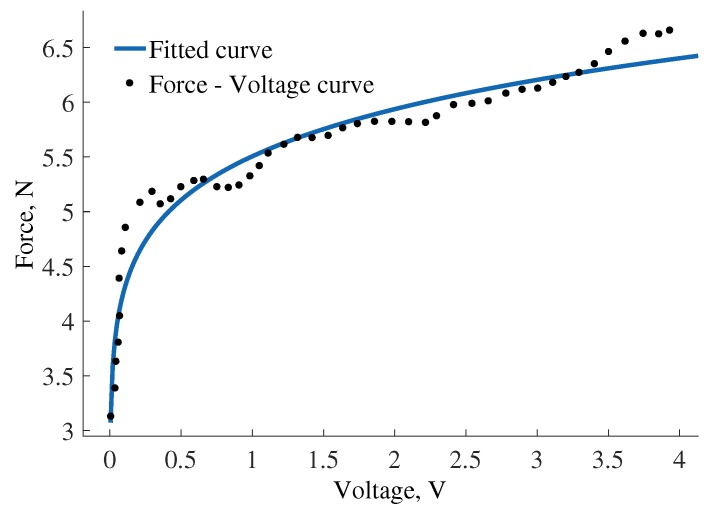
Calibration curve for tactile elements with spring rate 0.2 N/mm-fitted line and force-voltage response.

The error of fitting a spline to the measured force-voltage curve is calculated to evaluate the feasibility of the calibration. The best fitting curve was used to calculate the fitting error expressed as the coefficient of determination (R-squared). A good fit is considered for values above 0.95 for p<0.05. It was found that the R-squared value is less than 0.95 for the responses of tactile elements with spring rates 0.05 and 0.1 N/mm using polynomial and power fits, while the R-squared value for a spring rate 0.2 N/mm is 0.96 using first order power fitting. The fit is displayed in Figure 10. It can be observed that such sensor calibration provides good sensitivity for higher forces, namely for the range from 3 N to 6 N. In addition, we need to consider that the stiffness of the spring determines the lower threshold of the force, as the stiffer the spring is, the more force is required to compress it. To achieve higher force range for a spring with the limited length, as it is required for the design of miniature fingertips, we need to choose a relatively stiff linear spring that detects force from 3 N.

### 4.2. Sensor Performance Using an Ortho-Planar Spring

In this section, the response of the force tactile element with an integrated ortho-planar spring is tested separately; the linear spring is excluded from these experiments. In the previous section, based on the performance evaluation of the tactile element with a linear spring, it was decided to use a spring with a spring constant of 0.2 N/mm. The characteristic features of the tactile element with such spring lead to a good sensing range and small error of fitting due to calibration for forces from 3 to 6 N. As expected, the second spring of our hybrid tactile element, the ortho-planar spring, should be made particularly sensitive to small forces. This property is an important feature for the proposed fingertip sensor, as it is required to detect the forces during initial contact with an object. In addition, the measurement of small forces during grasping enables the understanding of whether the planned hand and finger configuration is adequate to implement a successful grasp, or if the finger was not positioned correctly.

The FE modeling results have demonstrated the force–displacement relationship for different parameters of the ortho-planar spring. Based on the evaluation of the force sensing range of the linear spring, the selected ortho-planar spring should measure forces below 3 N. Therefore, the focus is on the spring with leg thickness of 0.6 mm that has the maximum force range of 3.5 N (Figure 7). During the loading and unloading tests, it was found that the measurable force range of the ortho-planar spring is from 0.5 N to 3 N. As in our design the force is measured based on the displacement of mechanical components that leads to the modulation of light intensity, the appearance of static friction is unavoidable. This leads to the fact that we cannot measure force starting from zero, as it is shown in the FE simulations. This is inline with the results of FE analysis, as well as satisfying the requirements of the integrated sensing system. Next, it is observed how the tactile element with the ortho-planar spring is behaving for loading and unloading cycles. It was found that the the sensitivity of the force measurement is 0.5 V/N.

### 4.3. Performance of the Tactile Fingertip Using a Combined Spring System

**Figure 11:**
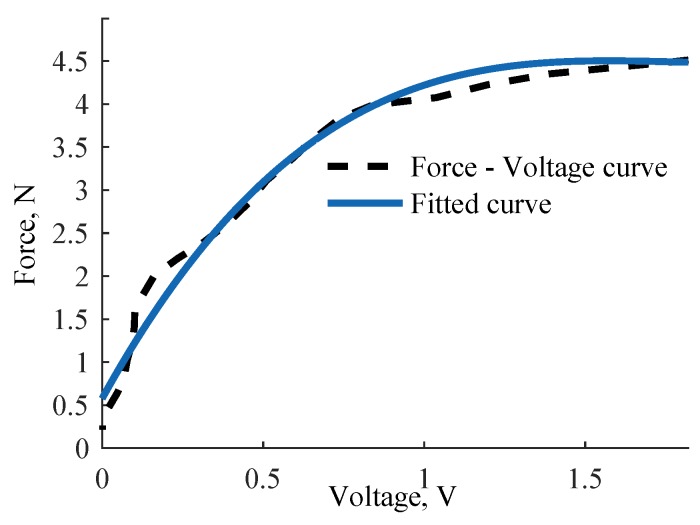
Calibration curve for a hybrid tactile element employing two springs: ortho-planar spring and linear spring. The two springs were chosen based on the described experimental study: the linear spring has a constant of 0.2 N/mm and the height of the leg of the ortho-planar is 0.6 mm.

Our hybrid approach to tactile sensing consists of two force sensing levels: an ortho-planar spring to measure small forces at the first level and a standard linear spring to measure higher forces at the second level. The first level is responding to light touch thanks to the integrated ortho-planar spring, which was chosen to be composed of legs with a thickness of 0.6 mm. The second level employing a linear spring with a spring constant of 0.2 N/mm has a wider sensing range but is less sensitive. Based on the evaluation of the combined response, the behavior is linear from 2.3 N to 4 N. A third order linear equation can be used to describe the force response for slightly larger forces. The fitted calibration curve of the behavior of the hybrid force sensing element is shown in Figure 11. Based on this calibration, the force sensing range is from 0.5 N to 4.5 N. When the sensor reaches 4.5 N, it is set to reach the saturation. Spring constants of linear and ortho-planar springs are selected to be different in order to measure different force ranges. Therefore, there is a slight overlap in the response that leads to the reduced force range when the springs are combined. The response of the sensor can be described with the following polynomial equation:

(6) $$F=0.7227V^{3}-3.859V^{2}+6.777V+0.5793.$$

With this study, we demonstrate the improved force behavior for a single hybrid tactile element of the multi-element fingertip sensor array. In the next section, the above configuration and respective calibration are used to evaluate the performance of the tactile array in the fingertip sensor during simple grasping tasks.

### 4.4. Evaluation of Tactile Array

In this section, we demonstrate the combined performance of a sensor array integrated with a fingertip. The array is composed of four tactile elements, as it is shown in Figure 1a,b. The proposed structure of the tactile sensing element allows for discriminating the types of objects grasped based on its stiffness. During grasping, the ortho-planar springs only provide feedback in a small force range. This type of grasp interaction is either a result of a low-force contact with a hard object during grasping or can occur from contact with a soft or light object. In case the sensor-equipped fingers are moved in order to achieve a tighter grasp, two main scenarios of force feedback can occur: (1) for the rigid object, the contact force is increased until the dome-shaped elements are fully pressed and good contact is established; and (2) the persistent light contact along with the motion of the finger shows that the object is either deflecting or is made from a soft material [25]. In our case, the force sensing element can be deformed up to 5 mm. This overall deformation includes the deflection of the ortho-planar spring (1 mm), as well as the compression of the linear spring. Therefore, the motion of the finger beyond this distance after initial contact had occurred results in a deflection or deformation of the grasped object. For rigid objects, the continued motion of a finger leads to higher contact forces until the moment the tactile element is fully depressed and reaching saturation.

**Figure 12:**
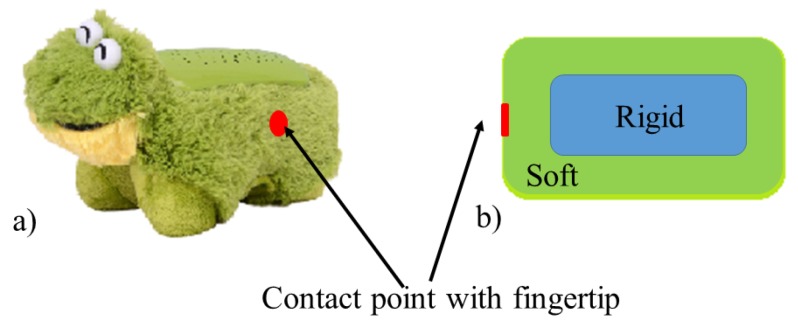
Photo and schematic representation of a toy object that acts as a benchmark to evaluate the performance of the integrated tactile array.

To evaluate the performance of a tactile array, a soft toy with a hard core was chosen. The toy and its schematic representation are shown in Figure 12. This type of object can be used to benchmark the performance of the integrated tactile array. The integrated fingertip was pressed against the toy object using a linear slide. In order to quantify the performance of the tactile array, a force / torque sensor was attached at the back of the fingertip. The fingertip and the sensor were fixed, while an object was moving towards the fingertip at a speed of 0.25 mm/s.

**Figure 13:**
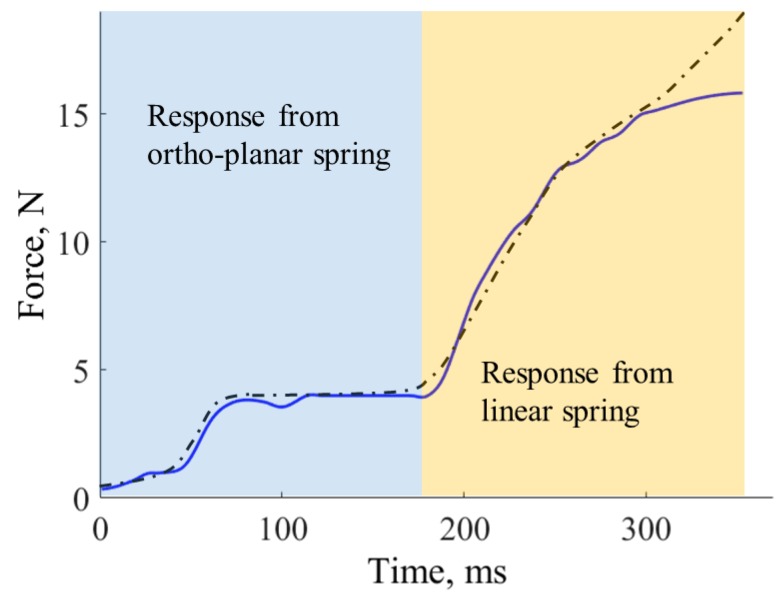
Force measurements from the fingertip sensor and force and torque sensor.

The response of the tactile array sensor is shown in Figure 13. The force response was calculated for each tactile element separately using Equation (6). Then, the single responses were added up to calculate the combined response for the tactile array. The graph clearly shows two-stage responses. Initially, the ortho-planar spring is compressed, then reaching saturation (flat stage in the graph). As soon as the soft part of the toy is deformed, the response from the rigid part is sensed. The combined sensing range of the tactile array is from 0.7 N to 16 N. The obtained force from the fingertip sensor was compared with the measurements from the force and torque sensor. The mean error across all measurement curves is 0.5 N.

## 5. Discussion

The presented work describes the design and parameters of an integrated tactile sensor array, and studies the performance of a single tactile element, which forms part of the array. The proposed single tactile element is based on a hybrid approach making use of a flexible ortho-planar spring to measure small forces and a standard stiffer linear spring to extend the measurement range to higher forces. The proposed approach is specifically targeted to miniature structures with space restrictions that use the light intensity modulations approach. The mechanical parameters of the springs were evaluated separately in order to find the best combination for the proposed sensing system. We have used a 3D printed ortho-planar spring in our studies, but it is likely that choosing other flexible materials might lead to the detection of even lower forces.

It was shown in Section 4.4 that the proposed sensing system is able to discriminate between soft and hard objects. In the future, we plan to implement tactile fusion algorithms for online grasping tasks. It is envisaged that these algorithms can significantly improve grasping across a broad range of objects, as well as improve the stability of a grasp.

The next stage of the prototype improvement process is further miniaturization of the prototype. It is planned to reduce the size of a single tactile element, and, at the same time, to increase the number of tactile elements per fingertip. The use of a tactile array with an increased spatial resolution allows for detecting points of contact with an object more accurately.

## 6. Conclusions

Beyond grasping, the proposed sensing method based on fiber-optical technology can be widely applied in various fields of engineering and automation. Equipping articulated mechatronic devices with tactile sensors leads to improved physical interaction with the environment with applications in human–machine interaction, manufacturing, healthcare, etc. It is important to highlight that the sensor can be fabricated cheaply owing to the low cost of the employed manufacturing approach, based on optic fibers and rapid prototyping, paving the way for an easy integration with robotic systems.

## Figures and Tables

**Table 1:** Characteristics of tactile elements using different linear springs.

Spring Rate, N/mm	Maximum Force, N	Sensitivity, V/N	Resolution, N
0.05	1.1	2.85	0.003
0.1	1.7	2.70	0.004
0.2	6.6	0.28	0.036
